# A Novel Testing Approach for Oxidative Degradation Dependent Incompatibility of Amine Moiety Containing Drugs with PEGs in Solid-State

**DOI:** 10.3390/pharmaceutics12010037

**Published:** 2020-01-02

**Authors:** Blaž Robnik, Katerina Naumoska, Zdenko Časar

**Affiliations:** 1 Lek Pharmaceuticals d.d., Sandoz Development Center Slovenia, Verovškova ulica 57, SI-1526 Ljubljana, Slovenia; blaz.robnik@sandoz.com; 2 University of Ljubljana, Aškerčeva cesta 7, Faculty of Pharmacy, Chair of Medicinal Chemistry, SI-1000 Ljubljana, Slovenia; 3 Department of Food Chemistry, National Institute of Chemistry, Hajdrihova 19, SI-1001 Ljubljana, Slovenia; katerina.naumoska@ki.si

**Keywords:** reactive excipient impurities, polyethylene glycol (PEG), iron oxide (Fe_2_O_3_), compatibility studies, stress testing, *N*-methylation, *N*-formylation

## Abstract

Reactive impurities originating from excipients can cause drug stability issues, even at trace amounts. When produced during final dosage form storage, they are especially hard to control, and often, factors inducing their formation remain unidentified. Oxidative degradation dependent formation of formaldehyde and formic acid is responsible for *N*-methylation and *N*-formylation of amine-moiety-containing drug substances. A very popular combination of polyethylene glycols and iron oxides, used in more than two-thirds of FDA-approved tablet formulation drugs in 2018, was found to be responsible for increased concentrations of *N*-methyl impurity in the case of paroxetine hydrochloride. We propose a novel testing approach for early identification of potentially problematic combinations of excipients and drug substances. The polyethylene glycol 6000 degradation mechanism and kinetics in the presence of iron oxides is studied. The generality of the proposed stress test setup in view of the susceptibility of amine-moiety-containing drug substances to *N*-methylation and *N*-formylation is evaluated.

## 1. Introduction

Polyether compounds such as polyethylene glycol (PEG), polyethylene oxide (PEO), and poloxamer are well-established pharmaceutical excipients with a low level of toxicity and potential use in a wide range of medical applications [1]. If stored appropriately, polyether compounds can show very good stability, even at extreme temperatures [2]. At the same time, they are very susceptible to degradation by molecular oxygen, due to the labile nature of protons at α-carbon atoms, and they can also degrade vastly at lower temperatures under oxidative conditions [3,4]. Their degradation mechanism at elevated temperatures has been extensively studied through chain length distribution changes [3,5,6], the characterization of small molecule degradation products [4,6,7,8,9], and theoretical kinetic modeling [10]. Chain scission is proposed as the predominant degradation mechanism, which results in the formation of shorter chain polymers and multiple small molecular weight organic impurities. Destabilization factors and catalysts affecting polyether degradation within pharmaceutical dosage forms have also been studied [11,12,13,14]; however, some interactions are still not understood completely. For the most often used polyether in solid dosage forms, PEG, there are several reports of destabilization events in solid dosage forms. These events are either related to PEG phase separation [12,15] or the presence of a trace amount of residual impurities such as reactive oxygen species [16] and transition metals [17,18], which can be present in raw materials and are known to promote degradation [19]. On the other hand, transition metals can also be purposely added to the formulations. Iron oxides are one of the most often used colorants, and their use is becoming increasingly important due to regulations affecting some synthetic organic dyestuffs [1]. Iron oxides are commonly used in conjunction with PEG as part of colored film coatings for tablets. Out of 59 FDA drug approvals in the year 2018, 23 products were in tablet form and more than two-thirds (15) contained a combination of PEG and iron oxide [20,21].

Formaldehyde and formic acid are predominant reactive small organic impurities originating from PEG degradation. Their reactions, in particular with amines, have been extensively studied [22,23,24,25], also in relation to pharmaceutical drug substances [11,12,13,26,27,28,29,30]. Formic acid is frequently responsible for the formation of *N*-formyl impurities from drug substances that contain primary or secondary amino moieties [12,15,30]. On the other hand, *N*-methyl impurities are formed in an Eschweiler–Clarke reaction, where both formaldehyde and formic acid are required to assure reaction progress [12,25,31]. A reliable and convenient screening method allowing scientists to predict the oxidative stability of drug substances against these reactions is lacking. Predicting the oxidative instability of drug substances in a reliable and meaningful manner has remained a challenge for the pharmaceutical industry [32]. Understanding the type and degree of degradation to which a drug candidate might be susceptible to in a solid dosage form is paramount in the early stages of drug development. However, to some extent, there is a lack of awareness of the oxidants and catalysts responsible for the oxidation of components in heterogeneous solid state system [33]. As drug substance-excipient incompatibility studies are commonly performed as binary mixtures, often only with components that are in direct contact, certain interactions might be overlooked [34,35]. Likewise, a multicomponent mixture would not be capable of identifying all potential interactions, since it does not adequately resemble environmental conditions established in the solid dosage form. Typically, drug development scientists decide to “spike” the drug substance with a reactive impurity directly, i.e., with solutions of formic acid, formaldehyde, or hydrogen peroxide. This approach has been met with criticism and is often found less suitable for solid state stability predictions, since the inherent excipient impurities are expected to be in a molecularly different form (such as formate esters instead of free formic acid or organic peroxides instead of hydrogen peroxide) [7,36,37]. Furthermore, such tests are very specific and can only test for one reactive impurity per test. More often than not, these reactive impurities are also toxic, allergenic, carcinogenic, or mutagenic [38,39].

**Figure 1:**
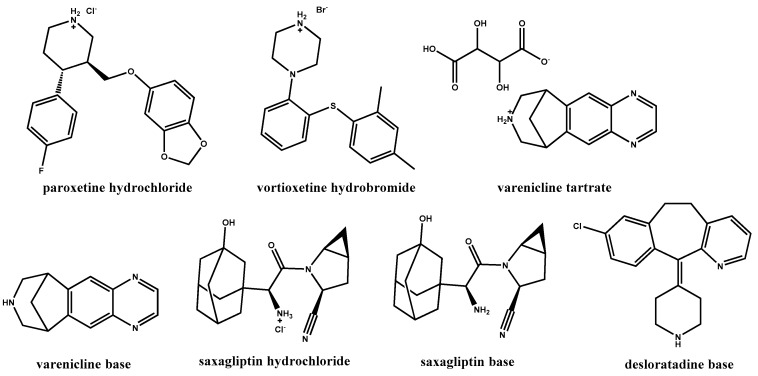
Model amine functionality containing drugs included in the study.

In this study, our objective is to design and apply an oxidative stressing procedure, which aims to create the correct oxidant, with the use of actual excipients and actual ratios from the solid dosage form [40,41,42]. We study the factors affecting degradation kinetics of one of the most frequently used PEGs for tablet formulations, PEG 6000 [1,21,43,44], in the presence of iron oxides in relation to the reactivity of paroxetine hydrochloride, a secondary amine functionality-containing drug substance [45], against *N*-methylation. The underlying factors required for this degradation pathway to be operative, including the involvement of iron oxides, are evaluated. *N*-methylation versus *N*-formylation degradation kinetics are also discussed. The generality of the developed incompatibility stress test is assessed through additional six compounds containing amine functionality (presented in Figure 1).

## 2. Materials and Methods

### 2.1. Materials

The reagents used for the analysis were hydrochloric acid (HCl) Titrisol^®^ solution, acetic acid (glacial) 100% anhydrous, ammonia solution 25%, ammonium acetate, tert-butyl methyl ether (MTBE), *O*-phosphoric acid 85%, and pyridine, 1-ethyl-3-(3’-dimethylaminopropyl)carbodiimide·HCl (EDC) from Merck KGaA (Darmstadt, Germany); acetonitrile (ACN) from J.T. Baker (Radnor, PA, USA); methanol (LC-MS grade) and ammonium bicarbonate (LC-MS grade) from Honeywell (Sellze, Germany); and 2-nitrophenylhydrazin HCl (NPH) from Tokyo Chemical Industry Co., Ltd. (Tokyo, Japan). Additionally, purified water used for the analysis was obtained by a Milli-Q gradient system from Merck Millipore (Burlington, MA, USA). Certipur^®^ buffer solutions were used for the pH calibration of pH meters from Merck KGaA (Darmstadt, Germany). All other reagents were of analytical grade.

The materials used for stress testing were povidone K30 (polyvinylpyrrolidone, PVP) from BASF (Ludwigshafen, Germany), polyethylene glycol (PEG) 6000 from Clariant (Burgkirchen, Germany), yellow and red iron oxides (Fe_2_O_3_) from Venator (Torino, Italy), triethyl citrate (TEC) from Vertellus Specialty Materials (Greensboro, NC, USA), Quinoline yellow lake from Colorcon (West Point, PA, USA), and hydroxypropyl methyl cellulose (substitution type 2910) (HPMC) from Shin-etsu Chemical Co., Ltd. (Tokyo, Japan).

Other equipment and materials used for analysis were a Centrifuge Eppendorf 5804 R, Eppendorf (Hamburg, Germany), Amicon Ultra-4, Ultracel^®^ 3K ultrafilters (Merck KGaA, Darmstadt, Germany), a Handy step^®^ electronic repetitive pipette (Brand GmbH, Wertheim, Germany), Biohit Picus pipettes (Sartorius, Göttingen, Germany), a Thermomixer comfort 5355 (Eppendorf, Hamburg, Germany), a PTFE membrane filter (Merck Millipore, Carrigtwohill, Ireland), a hydrophilic polytetrafluoroethylene (H-PTFE) filter (Macherey-Nagel GmbH & Co. KG, Düren, Germany), and a Millex-GV Syringe Filter Unit, 0.22 µm, PVDF (Merck KGaA, Darmstadt, Germany).

### 2.2. Purchased Standards of Drug Substances and Impurities

The purchased standards were paroxetine hydrochloride hemihydrate (purity of 99.9%), *N*-methyl paroxetine (purity of 100%), desloratadine (purity of 99.8%) and *N*-formyl desloratadine (purity of 99.0%), and these were purchased from the United States Pharmacopeial Convention (North Bethesda, MA, USA). Vortioxetine hydrobromide (purity of 99.9%) was obtained from Sandoz International GmbH (Holzkirchen, Germany). *N*-Formyl vortioxetine (purity of 100%), varenicline (purity of 97.2%), *N*-formyl varenicline (purity of 100%), *N*-methyl varenicline (purity of 98.9%), and *N*-formyl saxagliptin (purity of 99.7%) were purchased from TLC Pharmaceutical Standards (Newmarket, ON, Canada). Varenicline tartrate (purity of 98.2%) was purchased from Hangzhou Dayangchem Co., Ltd. (Hangzhou, China). *N*-methyl desloradatine (purity of 96.0%) was purchased from Toronto Research Chemicals (Toronto, ON, Canada). Saxagliptin·HCl dihydrate (purity of 99.5%) and saxagliptin monohydrate (purity of 99.5%) were purchased from MSN Pharmachem Pvt. Ltd. (Hyderabad, India). Low molecular weight organic impurity standard formic acid (purity of 99.8%) and formalin (37.0% formaldehyde solution) were obtained from Merck KGaA (Darmstadt, Germany).

### 2.3. Synthesized Standards of Impurities

For *N*-formyl paroxetine synthesis, paroxetine·HCl (1.02 g) was dissolved in 50 mL of water and the pH was adjusted to 9 with sodium carbonate. After extraction into ethyl acetate, the solution was dried (magnesium sulfate) and filtered and the solvent was evaporated in a rotary evaporator to obtain 0.89 g of solid. To 0.61 g of obtained material, 2 mL of ethyl formate (13 molar equivalents) was added, and the mixture was stirred and heated at reflux (65 °C) for 3 h. The mixture was dissolved in ethyl acetate and spotted onto an aluminum–silica TLC plate with a fluorescent indicator (F_254_). Spots at *R*_F_ ~ 0.7 and *R*_F_ ~ 1.0 were obtained. Both fractions were isolated on an Isolera^TM^ Prime flash chromatography automated system (Biotage, Uppsala, Sweden) using a Biotage^®^ SNAP 50 G column, methylene chloride (80%) as a weak solvent, and methanol (20%) as a strong solvent. The liquid chromatographic-mass spectrometric (LC-MS) method (Supplementary Materials) showed single peak for the isolated fraction with R_f_ ~ 1.0 with *m/z* = 358, which corresponds to *N*-formyl paroxetine (Supplementary Materials). A quantity of 0.47 g of yellowish resin was obtained after solvents were evaporated with a rotary evaporator. ^1^H and ^13^C NMR recorded spectra confirmed the structure of *N*-formyl paroxetine (Supplementary Materials). An NMR purity of 91% was determined on an as is basis.

*N*-methyl vortioxetine synthesis. Vortioxetine·HBr (0.50 g) was dissolved in 50 mL of water and the pH was adjusted to 10 with sodium carbonate. After extraction into ethyl acetate, the solution was dried (magnesium sulfate) and filtered and the solvent was evaporated in a rotary evaporator to obtain 0.37 g of solid. A quantity of 123.5 mg of obtained material was dissolved in 20 mL of methylene chloride to which 67.7 mg of trimethyloxonium tetrafluoroborate (1.05 molar equivalents) and 250 µL of *N*,*N*-diisopropylethylamine (3.5 molar equivalents) were added, and the mixture was stirred at room temperature under an inert atmosphere for 3 h. The reaction mixture was spotted onto an aluminum–silica TLC plate with a fluorescent indicator (F_254_), and spots at *R*_F_ ~ 0.67 and *R*_F_ ~ 0.83 were obtained. Both fractions were isolated on an Isolera^TM^ Prime flash chromatography automated system (Biotage, Uppsala, Sweden) using a Biotage^®^ SNAP 50 G column, methylene chloride (90%) as a weak solvent, and methanol (10%) as a strong solvent. The LC-MS method (Supplementary Materials) showed a major peak for the isolated fraction with *R*_F_ ~ 0.83 with *m/z* = 313, which corresponds to *N*-methyl vortioxetine. A quantity of 70 mg of white solid was obtained after solvents were evaporated with a rotary evaporator. ^1^H and ^13^C NMR recorded spectra confirmed the structure of *N*-methyl vortioxetine (Supplementary Materials). An NMR purity of 79% was determined on an as is basis.

*N*-methyl saxagliptin synthesis. Saxagliptin·HCl (2.01 g) was dissolved in 10 mL of water and the pH was adjusted to 10 with sodium carbonate. After extraction into ethyl acetate, the solution was dried (magnesium sulfate) and filtered and the solvent was evaporated in a rotary evaporator to obtain 0.76 g of solid. A quantity of 360.0 mg of obtained material was dissolved in 20 mL of methylene chloride to which 167 mg of trimethyloxonium tetrafluoroborate (1.05 molar equivalents) and 350 µL of *N*,*N*-diisopropylethylamine (2.05 molar equivalents) were added, and the mixture was stirred at room temperature under an inert atmosphere for 3 h. The reaction mixture was flushed with water and the organic phase was separated, dried (magnesium sulfate), and filtered. The LC-MS method (Supplementary Materials) showed a major peak with *m/z* = 330, which corresponds to *N*-methyl saxagliptin. A quantity of 40 mg of white solid was obtained after solvents were evaporated with a rotary evaporator. ^1^H and ^13^C NMR recorded spectra confirmed the structure of *N*-methyl saxagliptin (Supplementary Materials). An NMR purity of 89% was determined on an as is basis.

### 2.4. Analytical Methods

#### 2.4.1. Determination of Drug Substances and *N*-methyl and *N*-formyl Degradation Products

An Acquity UPLC BEH C18 reverse phase column (100 × 2.1 mm; 1.7 µm, Waters Corporation Milford, MA, USA) connected to a 0.5 μm in-line filter (Idex, Health & Science, Oak Harbor, WA, USA) was used to quantify paroxetine, desloratadine, vortioxetine, varenicline, saxagliptin, and the corresponding *N*-methyl and *N*-formyl impurities using a UHPLC-MS system (Dionex Ultimate 3000—LCQ Fleet, Thermo Fisher Scientific, San Jose, CA, USA). Mobile phases A (10 mM ammonium bicarbonate buffer, pH 10.77) and B (methanol) were filtered through a 0.1 µm PTFE membrane filter. For paroxetine, desloratadine, and vortioxetine samples, isocratic elution of the mobile phase (20% A and 80% B), with a flow rate of 0.2 mL/min and a run time of 30 min was used. For varenicline and saxagliptin samples, the gradient elution program employed was as follows: 40% B (0–5 min), 40%–80% B (5–10 min), 80% B (10–30 min), 80% B–40% B (30–31 min), 40% B (31–40 min). The injection volume for all samples was 5 µL. Autosampler and column temperatures were maintained at 10 and 30 °C, respectively. Electrospray ionization in positive mode ((+)ESI-MS) was used to ionize the compounds. MS spectra were acquired in the *m/z* range of 100–1000. Quantification was performed using selected ion monitoring (SIM) acquisitions at *m/z*, as shown in Table 1. The MS conditions used for individual drug substances were optimized and are shown in Table 2.

Stock solutions of standards and samples were prepared in a concentration of about 1 mg/mL in methanol (paroxetine, desloratadine, vortioxetine) or in 60% methanol (varenicline and saxagliptin). Drug substance standards were further diluted with the corresponding solvent to obtain seven working solutions for plotting a calibration curve with the following concentrations (35, 30, 25, 20, 15, 10, and 5 µg/mL). Standards of *N*-methyl and *N*-formyl impurities were first diluted to 25 and 10 µg/mL and, subsequently, to ten additional working solutions for plotting the calibration curves with the following concentrations (3000, 1500, 750, 375, 187.5, 93.7, 46.9, 23.4, 11.7, 5.8 ng/mL). Stock solutions of the dissolved samples were filtered through a 0.20 µm H-PTFE filter. Sample stock solutions were further diluted with the same solvent to obtain working solutions with a concentration of 25 µg/mL (or 50 µg/mL for saxagliptin set). Dilutions of samples containing mixtures of drug substances and excipients were corrected for the percentage of added excipients. Method verification was performed and performance parameters are summarized in Table S1, Supplementary Materials. For determination of the paroxetine drug substance and *N*-methyl paroxetine impurity, the supplementary UHPLC-UV analytical method was used (Supplementary Materials).

#### 2.4.2. Determination of Low Molecular Weight Organic Impurities

The low molecular weight organic impurities formaldehyde and formic acid were determined by an adopted liquid chromatography method [15].

### 2.5. Stress Test Setup and Conditions

**Figure 2:**
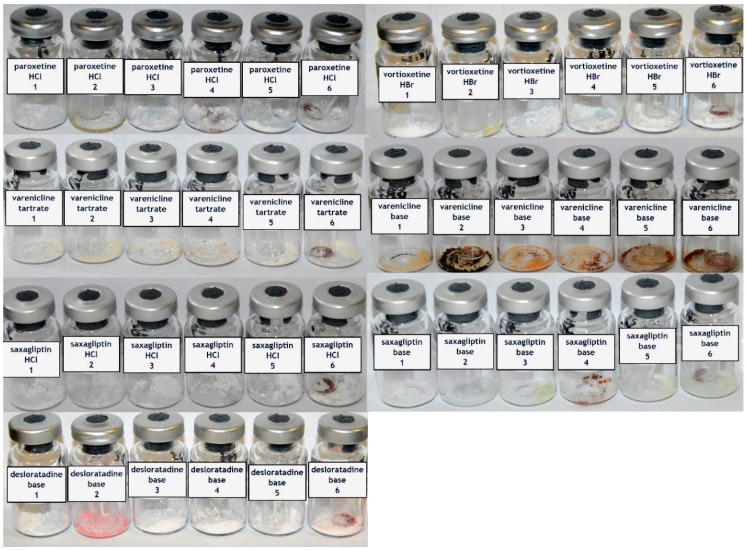
Photographs of samples of seven selected drug substances (137 µmoles of each) after a seven day stress test at 60 °C completion. Drug substances were evaluated alone (1), in combination with formalin in a glass insert (10 µL of 37.0% formaldehyde solution, equivalent to 134 µmoles of formaldehyde) (2), polyethylene glycol (PEG) 6000 (0.83 µmoles of PEG 6000, equivalent to 113 µmoles of ethylene glycol units) (3), and a mixture of PEG 6000 and iron oxide (Fe_2_O_3_) (6.3 µmoles of Fe_2_O_3_, equivalent to 3.1 µmoles of Fe^2+^ ions) (4). Novel compatibility setups (5) and (6) included an additional glass insert inside the 10 mL vial, where PEG 6000 (5) or a mixture of PEG 6000 and Fe_2_O_3_ (6), were held separate from drug substances.

Approximately 136.68 µmoles of each drug substance, equivalent to 50.0 mg of paroxetine·HCl, was weighed into 10 mL glass vials and closed with rubber silicon stoppers and aluminum pull-tab seals. The closed design was utilized to best mimic the actual conditions of packed film-coated tablets. Lyophilisator Scanvac Coolsafe from LaboGene (Lillerød, Denmark) was used for flushing the samples with nitrogen from Messer GmbH (Krefeld, Germany). The incubator binder BF 729 from Binder GmbH, Tuttlingen, Germany was used for stability testing. The temperature of the stress test was set to 60 °C, close to the melting point of PEG 6000, to accelerate degradation processes, but not high enough to significantly influence oxidative degradation mechanisms [4]. The test duration was seven days. Different stress test setups were evaluated. Excipient mixtures, such as PEG 6000 and Fe_2_O_3_, were pre-mixed with the Thinky ARE-250 planetary mixer from Intertronics (Oxfordshire, UK) to attain good contact between components. The final compatibility setup included additional glass inserts inside the 10 mL vial, where excipients or mixtures of excipients were held separately from direct contact with paroxetine·HCl. Samples of seven drug substances after stress test completion are presented in Figure 2.

### 2.6. Kinetic Model and Statistical Evaluation

A set of ordinary differential equations was solved in the Berkeley Madonna^®^, version 8.0.1, mathematical modeling software package (University of California, Berkeley, CA, USA). The runge–kutta (fourth order) integration algorithm was used with a tolerance of 0.00001. Nonlinear regression analysis was performed by the Levenberg–Marquardt algorithm. Standard deviation errors for fitted kinetic constants were determined with Phyton programming package through curve fit optimization routine. Interaction plots and other statistical analysis were performed with Minitab statistical software program (Minitab, LLC, State College, PA, USA).

## 3. Results and Discussion

### 3.1. PEG Incompatibility Stress Test: Paroxetin·HCl

**Scheme 1:**
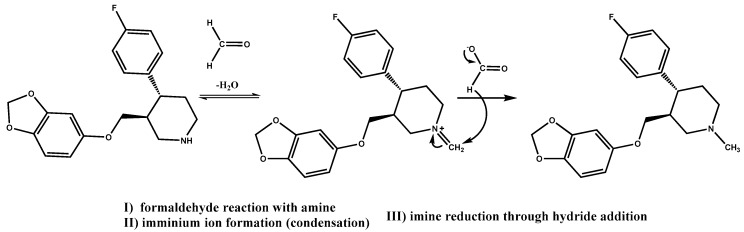
Paroxetine to *N*-methyl paroxetine degradation reaction [25,31].

In a recent drug development effort in our laboratories, paroxetine·HCl [45], a drug substance containing a secondary amine moiety, was formulated as a tablet. It was found that a significant amount of *N*-methyl paroxetine impurity, a product of an Eschweiler–Clarke reaction (Scheme 1) [25,31], was formed in packed tablets protected from moisture and light under accelerated stability conditions (data not reported here). The tablet formulation consisted of (1) tablet core components—paroxetine·HCl, hypromellose 2208 (hydroxypropyl methylcellulose, HPMC), povidone K30 (polyvinylpyrrolidone, PVP), lactose monohydrate, silicon dioxide, magnesium stearate; (2) the enteric coat components—methacrylic acid and ethyl acrylate copolymer, talc, triethyl citrate; and (3) the over coat components—hypromellose 2910 (hydroxypropyl methylcellulose, HPMC), polyethylene glycol (PEG) 6000, titanium dioxide, and iron oxide (Fe_2_O_3_) yellow. An investigation to determine the origin of formaldehyde and formic acid responsible for *N*-methylation was conducted.

#### 3.1.1. Origin of Reactive Impurities in Tablet Formulation

Tablet composition components with the potential to contain trace amounts of reactive impurities, were studied in the form of binary and tertiary mixtures with the drug substance in a sealed vial setup. Povidone K30, due to its direct contact with paroxetine·HCl, as well as triethyl citrate (TEC) and PEG 6000, found in the tablet coat, were considered for testing in a direct mixture setup. Surprisingly, when subjected to 60 °C for seven days, none of these mixtures resulted in an increase in *N*-methyl paroxetine (Table 3, Experiment 1). In the next step, we added Fe_2_O_3_ to PEG 6000 as a potential oxidation catalyst and constructed a direct and separate mixture design. In a separate mixture, design excipients were transferred into glass inserts within the large vials containing paroxetine·HCl. This setup was introduced to best mimic the conditions in the tablet formulation, where there is also no direct contact between components. Interestingly, the reaction for *N*-methyl paroxetine was positive, but only where PEG 6000 and Fe_2_O_3_ are separated from paroxetine·HCl (Table 3, Experiment 2). Tablet core excipients were first thought to be responsible for the impurity increase; therefore, such an outcome was unexpected. Similarly to tablet stability results, no other paroxetine degradation was observed, indicating a very good resemblance of this stress test compared to the actual conditions within tablets. Based on the stress test results, it is hypothesized that volatile low molecular weight organic impurities originating from oxidative degradation of plasticizer PEG 6000 in contact with Fe_2_O_3_ permeated from the tablet coat into the tablet core through the enteric coating and concurrently reacted with the drug substance. The same stress test design was applied to an alternative plasticizer and alternative colorant (Table 3, Experiment 3). Both results were negative, which confirmed that the combination of PEG and Fe_2_O_3_ is accountable for incompatibility with the drug substance. After 14 days, the level of *N*-methyl paroxetine increased from 0.63% to 0.84% in the sample with PEG 6000 and Fe_2_O_3_ yellow; in other samples, this increase did not occur.

To confirm that the cause of *N*-methylation are in fact reactive impurities formaldehyde and formic acid, paroxetine·HCl in a solid state was spiked with formalin, a 37% formaldehyde solution, and with 99.8% formic acid, prior to elevated temperature exposure. All instances of formalin addition, direct and in separate insert, resulted in *N*-methyl paroxetine impurity formation, on the contrary to formic acid addition, which is not capable of forming *N*-methylated product (Table 4). Nonetheless, repeatability of the results with reactive impurity spiking is poor. The determined *N*-methyl paroxetine amounts for repetitions of formalin added to paroxetine·HCl showed extensive variability (average: 0.98%, rsd: 76%, *n* = 3) and poor specificity, with multiple by-products formed and high drug substance degradation. In practice, it is very challenging to add low volumes of reactive impurities in an accurate and precise manner. What is more, added liquids cannot be homogeneously distributed across the entire sample, regardless of whether they are added directly to the sample and mixed or if they are in a separate container, which presents sampling issues. Reactive impurities solutions often also contain other impurities. Formalin for instance contains methanol, formic acid, aldehydes, and ketones, which have an influence on the outcome of the experiment. On the other hand, combination of PEG and Fe_2_O_3_, separated from paroxetine·HCl, resulted only in *N*-methyl impurity formation (more than 99% of drug substance remaining), with lower variability (average: 0.63%, rsd: 13%, *n* = 3). For more in-depth understanding of PEG degradation and iron oxide involvement in *N*-methylation of paroxetine, a kinetics study was planned.

#### 3.1.2. PEG Degradation and *N*-Methyl Formation Kinetic Study

**Scheme 2:**
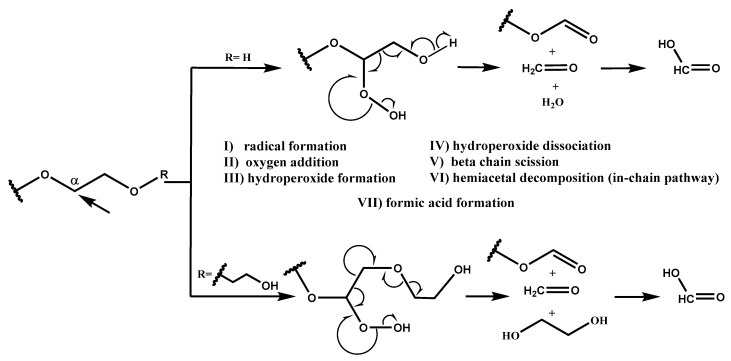
Example of radical chain scission at the terminal (upper pathway) and penultimate (bottom pathway) glycol units of polyethylene glycol (PEG) [3,14]. Carbon atom subjected to oxygen attack is annotated with α.

Based on the radical chain scission mechanism (Scheme 2) [3,14], very low concentration of radical sites is needed to initiate the PEG degradation process. These sites can form under the influence of heat, near-UV light, chemical initiators, or transition metals (step I) [7]. In the propagation phase (steps II, III, IV, V, and VI), first the addition of oxygen occurs. PEG requires molecular oxygen to degrade into formaldehyde and formic acid. Then, peroxy group abstracts hydrogen from a nearby RH, and this is followed by hydroperoxide dissociation and alkoxy radical (RO·) formation, resulting in C–C bond scission and, finally, the formation of formaldehyde and formate ester [10], which can hydrolyze into formic acid. In-depth NMR studies performed by Mkhatresh et al. [5,8] identified, apart from formaldehyde and formate ester end-groups, also the following species: In-chain esters, methyl end-groups, ethanoate end-groups, acetal links, and multiple small molecular species. Gallet et al. added to these lists also ketones and alcohols [3,4]. Nevertheless, our study design focused on monitoring formaldehyde and formic acid, as the main PEG degradation products. Each ethylene glycol unit in theory can produce one formaldehyde molecule, which can react with amine functionality of paroxetine. Proposed amounts and ratios of components in 10 mL glass vials (i.e., 1:0.1:0.01), present sufficient reactive reactant availability on one hand, since 68.6% of *N*-methylated impurity at complete conversion is formed, and realistic excipient-drug substance ratio on the other.

**Figure 3:**
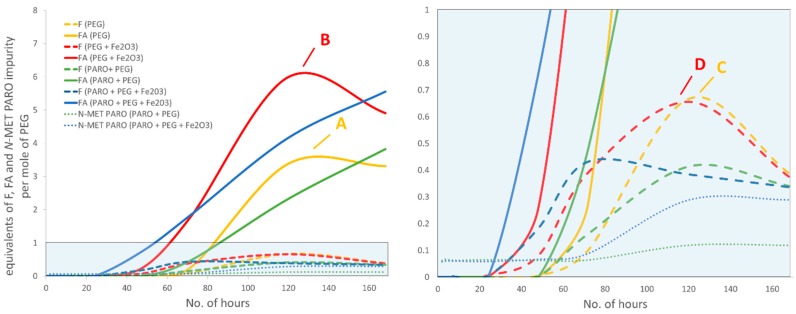
Polyethylene glycol (PEG) and paroxetine (PARO) degradation kinetic study. The monitored degradation products were as follows: Formaldehyde (F), depicted with dashed lines, formic acid (FA), depicted with solid lines, and *N*-methyl paroxetine (*N*-MET PARO), depicted with dotted lines. Samples with PEG only (yellow lines), PEG with iron oxide (Fe_2_O_3_) (red lines), PARO and PEG in separate glass inserts (green lines), and PARO and PEG with Fe_2_O_3_ in separate glass inserts (blue lines) were subjected to stress testing at 60 °C for up to seven days in closed vials. Points C, D and A, B indicate the maximum formaldehyde and formic acid concentrations, respectively. The time in hours is presented on the x axis, the equivalents per mole of PEG are presented on the y axis. The left graph represents the whole y-axis range, while the right graph represents an enlarged y-axis section.

In our kinetic study we paid special attention to events occurring in the early hours of the stress test, to evaluate potential formaldehyde to formic acid conversion and study the lag time between PEG and drug substance degradation. Results of the seven-day stress test study are presented in Figure 3.

**Figure 4:**
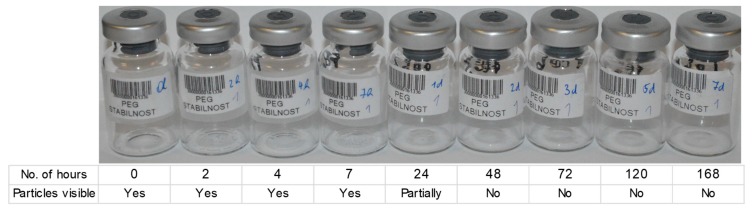
Samples of polyethylene glycol (PEG) only in glass vials after exposure to stress testing at 60 °C for up to seven days.

In the first 24 h, no increase in impurities was observed. This is in accordance with Bergh et al. who also observed some lag time prior to formaldehyde detection [9]. The lag time in our case correlated with the onset of melting. PEG 6000 is a solid powder with a melting point of 55–63 °C [1]. Visual inspection of samples revealed that solid particles could be observed for up to 24 h at an elevated temperature but were no longer visible after 48 h (Figure 4). At this time, a smooth film formed at the bottom of the vial. This indicates that degradation kinetics are connected to the mobility of PEG chains and oxygen molecules and are significantly increased when the sample is converted to melt. It is possible that transition to a liquid state is observed only when the molecular weight of longer PEG chains is reduced, which causes a lag time in the melting onset.

The maximum formaldehyde and formic acid concentrations in samples with PEG alone were reached after five days (points A and C, Figure 3): 3.38 mg of formaldehyde per g of PEG, which is 0.68 equivalents per mole of PEG, and 26.3 mg of formic acid per g of PEG, which is 3.43 equivalents per mole of PEG. The maximum concentrations of formaldehyde and formic acid in samples with PEG and Fe_2_O_3_ were also reached after five days (points B and D, Figure 3): 3.29 mg of formaldehyde per g of PEG, which is 0.66 equivalents per mole of PEG, and 46.1 mg of formic acid per g of PEG, which is 6.01 equivalents per mole of PEG. This suggests that in the design where PEG is combined with Fe_2_O_3_, at least six molecules of formic acid are formed per PEG chain, which is one per every 23 ethylene glycol units (4.4%). After five days, the concentrations of both degradants began to decrease (Figure 3). Maximal observed levels were up to 10-fold higher compared to the levels determined in similar studies performed by other groups [14,46]. These differences are especially ascribed to the temperature difference, which, in our case, was purposely set at the PEG 6000 melting point and the time from the stress test start to sampling, since considerable curvature was observed.

The observed formation of formaldehyde was unproportioned to the formation of formic acid, which was present in significantly higher concentrations. A comparison of the curves (solid red and yellow line compared to dashed yellow and red line in a range from ca. one day three days, Figure 3) suggests that at least some, if not the majority, of formic acid is formed from rapid formaldehyde consumption [14]. This observation is also confirmed by our previously mentioned experiments (Section 3.1.1.), where a significant increase in *N*-methyl impurity was observed in direct spikes of formaldehyde solution, where no formic acid was added. The maximum formaldehyde concentration was at an approximately equal level with PEG alone compared to when iron oxide was added (compare points C and D in Figure 3); however, the formic acid concentration was much higher in the latter (compare points A and B in Figure 3). This means that iron oxide has to be responsible for the accelerated formation of formaldehyde; otherwise, the molar sum of these two impurities would be the same in both experiments. The concentration of formaldehyde (dashed yellow and red lines, Figure 3) and formic acid (solid yellow and red lines, Figure 3) in samples without paroxetine·HCl started to fall after a five-day time point. A decrease in formaldehyde was expected due to oxidation to formic acid, however, the formic acid decrease was not expected. The reactant availability was initially considered. In the 10 mL glass vials used for our stress tests, there was 93.75 µmoles of O_2_ molecules, calculated based on the ideal gas law. Five milligrams of PEG 6000 placed in every vial is equivalent to 0.833 µmoles of chains with a molecular weight of 6000 g/mol. Considering there is, on average, 136 ethylene glycol monomer units in PEG 6000, the amount of oxygen available would suffice for the degradation of 82.8% of all ethylene glycolic units. Thus, the concentration of oxygen is sufficient for oxidation to proceed. Formaldehyde, as second essential reactant, is also available as long as there are unoxidized ethylene glycol units left, unless the material itself or the environmental conditions within the vial change, so that the PEG degradation rate is affected. Potential causes for more substantial reduction of the PEG degradation rate could be associated with the change in relative humidity (RH), pH, molecular weight reduction of PEG chains, and oxidative changes to the PEG backbone, resulting in inactivity. To our knowledge, there is no reported evidence in the literature that a drop in pH or increase in RH, through formed molecules of organic acids and water, could significantly decrease the PEG degradation rate. On the other hand, material properties change, such as a decrease in the length of PEG chains [47] or some other oxidative transformation, could influence the degradation rate. However, one would expect maximum formic acid concentration for samples with PEG alone (point A, Figure 3) compared to PEG with Fe_2_O_3_ (point B, Figure 3) to reach the same level if they are conditioned by material properties or environmental conditions change. Yet, the maximum formic acid concentration could be dependent on the presence of iron oxides, whereas a decrease in the PEG degradation rate after a five-day time point is related to some other physical change.

In our opinion, it is more likely that formic acid will react further, forming a tertiary degradation product, e.g., formate ester [4,14], creating a system of three consecutive reactions. Formate esters are expected to have similar reactivity to formic acid; however, their mobility is restrained due to long residual PEG chains. Similar reactivity is also expected for tablet formulations with PEG present in the tablet coat. Formic acid in formate ester form would not be detected with our method for the determination of small molecular weight organic molecules due to the inability to react with an EDC condensing agent.

The relevance of the proposed three consecutive reaction systems was evaluated via kinetic modelling. The reaction stoichiometry is described in Equation (1). “r” represents the reaction rate (change in concentration over time), “k” represents the rate constant, and “c” represents the concentrations of individual species. Experimental results for formaldehyde (F) and formic acid (FA) in the sample with PEG 6000 alone were fitted to the model described in Equations (2)–(5) (lag time was disregarded).

(1) $$PEG\;\left(r_{1}\right)\;\overset{k_{1}\;}{\to }F\;\left(r_{2}\right)\overset{k_{2}\;}{\to }\;FA\;\left(r_{3}\right)\overset{k_{3}\;}{\to }\;Tert.\;deg.\;prod.\;\left(r_{4}\right)\;$$

(2) $$r_{1}=-\;k_{1}c_{PEG}$$

(3) $$r_{2}=k_{1}c_{PEG}-k_{2}c_{F}$$

(4) $$r_{3}=k_{2}c_{F}-k_{3}c_{FA}$$

(5) $$r_{4}=k_{3}c_{FA}$$

**Figure 5:**
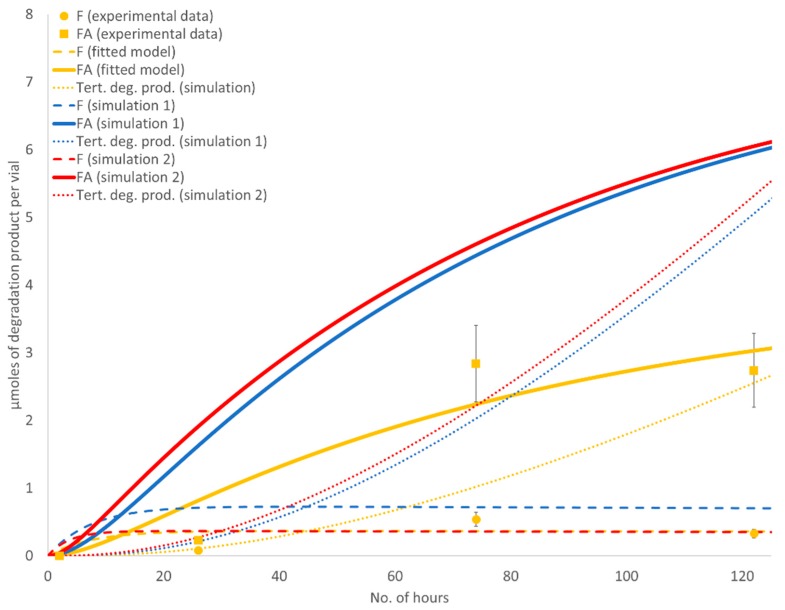
Experimental data, fitted model, and simulated model for polyethylene glycol (PEG) degradation kinetics. Experimental data for formaldehyde (yellow circles) and formic acid (yellow squares) concentrations in PEG only degradation samples, fitted model (yellow lines), and two simulations (blue lines—k_1_ was increased by 2-fold and red lines—k_1_ and k_2_, were increased by 2-fold) are depicted. Time in hours is presented on the x axis, µmoles of degradation product per vial are presented on the y axis. Abbreviations: Formaldehyde (F); formic acid (FA); hypothetical tertiary degradation product (tert. deg. prod.).

Figure 5 graphically depicts the PEG degradation kinetics of a model fitted to the degradation data of PEG alone (yellow lines). The determined rate constants for the “fitted model” were k_1_ = 4.32 × 10^−4^ ± 1.68 × 10^−4^ h^−1^, k_2_ = 0.134 ± 0.0730 h^−1^, and k_3_ = 0.0117 ± 0.00976 h^−1^, implicating fast formaldehyde to formic acid conversion (this explains the absence of lag time between both degradants) and an approximately 10-fold slower third consecutive reaction of tertiary degradation product formation from formic acid. Two hypothetical types of PEG degradation kinetics, where we simulated increases in rate constants, are depicted with red and blue lines. In “simulation 1”, the rate constant k_1_ was increased by 2-fold (blue lines), and in “simulation 2”, both, k_1_ and k_2_ were increased by 2-fold (red lines). It was previously established in our experiment that iron oxide has to be responsible for increasing k_1_ based on the increased molar sum of determined formaldehyde and formic acid in the sample with PEG and Fe_2_O_3_ (Figure 3). Simulations in Figure 5 show that if along with k_1_, k_2_ is also increased by the same factor, formic acid’s maximum is higher compared to the fitted model (solid red versus solid yellow), whereas maximum formaldehyde concentration is equal (dashed red versus dashed yellow line). Increasing only k_1_ results in an increased maximum formaldehyde concentration in the simulated model compared with the fitted model (dashed yellow versus dashed blue line). Since the simulated kinetics for increased k_1_ and k_2_ (red lines) reflect the behavior of the experiment depicted in Figure 3, we confirmed that the degradation of PEG to formaldehyde and formaldehyde to formic acid are both catalyzed by iron oxide. Formate ester formation, which could also influence PEG degradation kinetics by protecting terminal hydroxyl groups from oxidation, was not accounted for in the model [6].

Iron oxide increases the amount of formed *N*-methyl impurity through an increase in the PEG degradation rate and also through an earlier onset of small volatile PEG degradation products. Iron oxide is generally insoluble in water; however, only trace amounts are needed to trigger the formation of free radicals capable of initiating PEG chain degradation. Furthermore, PEG solutions are slightly acidic, which potentially increases the amount of free/dissolved iron in the sample. Transition metal ions may cause the formation of very reactive hydroxyl radicals in a well-known Fenton reaction [42,48], but there are also reports that they may act as direct oxidants in the absence of hydroperoxide impurities [49]. By means of radical formation, Fe^3+^ ions participate in formaldehyde oxidation and, in a similar way, in PEG oxidation. Another potential interpretation of an iron-oxide-induced increase in the degradation rate is through the formation of complexes. The oxidized form of the iron ion (Fe^3+^) is most likely to form complexes with oxygen and molecules containing oxygen, facilitating electron transfer [18]. Sakharov et al. suggested that catalytic oxidative deformylation, with Cu^2+^ as the catalyst, is the predominant degradation mechanism instead of radical chain scission [17]. The transition metal in this case does not carry a redox function but acts as a complexing agent, aiding in electron transfer. It is presumed that Cu^2+^ preferably forms complexes with longer PEG chains, which could be reflected by a decreased PEG degradation rate over time. Nevertheless, this mechanism is not the likeliest of the Fe mediated degradations, since extreme basic conditions are required.

When paroxetine, separated from excipient(s), was added to the samples, a sigmoid curve shape for formic acid concentration was no longer observed (solid blue and green lines, Figure 3). Faster initial formaldehyde and formic acid formation was associated with the smaller compartment where PEG was held, resulting in better heat transfer and an earlier onset of melting. Slower formation in the second part of the stress test was associated with the consumption of reactive impurities for the *N*-methylation reaction. The difference in formaldehyde concentration in samples with compared to those without paroxetine is in line with the concentration of formed impurities. However, it is also evident that not all formaldehyde reacts with paroxetine, and unexpectedly a high difference between *N*-formyl amounts in samples with and without Fe_2_O_3_ were observed (Figure 3, dotted blue versus green line). Based on the difference in levels of reactive impurities, this is ascribed to the critical concentration of formic acid needed for the reaction to proceed. Another reaction specific is plateau formation (Figure 3, dotted blue and green line). We believe that this plateau is related to two low concentrations of formaldehyde and formic acid in the vial. Equivalently, in the early phase of the stress test, formaldehyde and formic acid are already available, but the reaction with paroxetine does not start until they reach critical concentrations. Alternative interpretations for the plateau are (1) a drop in pH that would disable formic acid to release a proton and, consequently, hydride, (2) an increase in the concentration of water molecules that destabilizes the imine intermediate, or (3) an excessively high gas pressure, disabling carbon dioxide release. The paroxetine used in this experiment was already in salt form (hydrochloride); however, amine functionality protonation due to acid formation also decrease compounds’ nucleophilic properties and affects the reaction rate.

To conclude this evaluation, we compared the molar ratios of formed *N*-methyl impurities and the formaldehyde content in samples with paroxetine·HCl subjected to stress tests. A 100-fold higher molar ratio of *N*-methyl paroxetine to formaldehyde was observed in the experiment where the combination of neat PEG 6000 and Fe_2_O_3_ is stressed, compared with the direct addition of reactive impurities (i.e., formalin, 37% formaldehyde solution, Table 4), suggesting a more appropriate stressor for these types of reactions.

#### 3.1.3. Sensitivity of the Stress Test to Environmental and Micro-Environmental Factors

Additional experiments were run to evaluate dependence of *N*-methyl paroxetine formation on Fe_2_O_3_ concentration, the addition of solid diluent, solvents with different pHs, nitrogen atmosphere, or open-dish setup (Table 5).

Changes in Fe_2_O_3_ concentration are reflected in *N*-methyl paroxetine levels, however dependency is not linear. Due to the propagation step, already small amounts of Fe_2_O_3_ can result in significant PEG degradation and *N*-methyl paroxetine formation. On the other hand, at the higher concentrations of Fe_2_O_3_, a plateau is observed. Diluting the excipient mixture with HPMC was performed to emulate the environment in tablet formulation. Diluents were able to reduce the contact between iron oxide and PEG, and by this means, stop drug substance degradation. In a direct mixture of the drug substances, PEG and Fe_2_O_3_, the drug substance acts as diluent and prevents better contact of PEG with Fe_2_O_3_ (Table 3). Addition of water, through formation of water vapor, can hamper the formation of iminium ion in an equilibrium condensation reaction (Scheme 1). Water is also known to be able to decrease oxidation to some extent, for example, through peroxide decomposition [16]. Similarly, the addition of acid and alkali solutions also did not result in *N*-methyl paroxetine formation. Nitrogen purging completely stopped *N*-methylation, which is expected since oxygen is needed for PEG to degrade. Lastly, same is true for open-dish setup, where reactive impurities are able to evaporate out of the vial and do not come in contact with paroxetine.

### 3.2. Model Drug Substances with Amine Functionality Tested for PEG Incompatibility

Seven drug substances with amine functionality were selected to demonstrate the applicability of previous findings beyond the paroxetine example (Figure 1). Six samples for each of the seven compounds in evaluation were prepared (paroxetine included). Analysis of the drug substance alone (1), with the addition of formaldehyde in solution in a separate glass insert (2), in a direct mixture with PEG (3), in a direct mixture with PEG and Fe_2_O_3_ (4), with PEG in a separate vial (5) and with PEG and Fe_2_O_3_ in a separate glass insert (6) was performed after seven days at 60 °C in closed vials (Table 6). Our main objective was to assess the generality of the test setup, namely in terms of the potentiated *N*-methylation in the sample with a mixture of PEG and Fe_2_O_3_ without direct contact with the drug substance.

In addition, special attention was given to the sum of *N*-methylation and *N*-formylation, enabling us to rank the overall amine group reactivity against small molecular weight organic impurities and *N*-formylation versus *N*-methylation ratio to account for the reaction specificity (Table 7).

**Figure 6:**
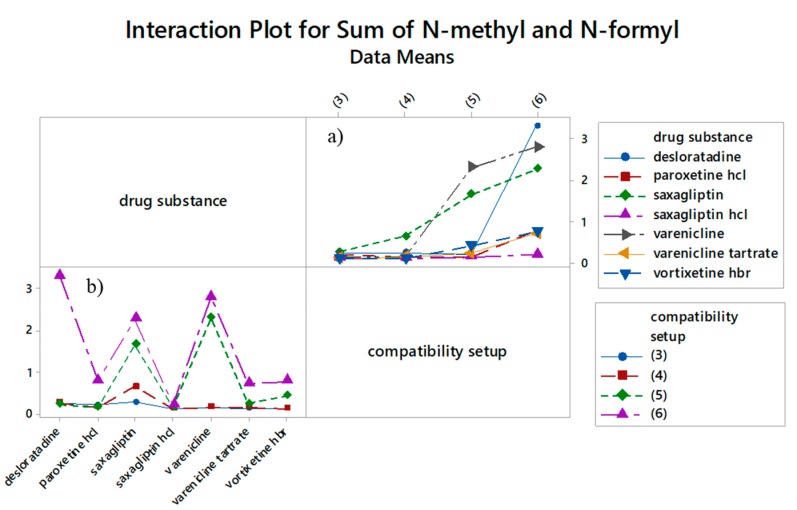
Interaction plot for sum of *N*-methyl and *N*-formyl impurities for different drug substances and compatibility setups. Data presented with ‘compatibility setup’ on x axis (**a**) and ‘drug substance’ on x axis (**b**).

Spiking of samples with a formaldehyde solution resulted in vast degradation and does not reflect the actual tablet formulation conditions well (assay decrease up to 80%—data not presented). Direct mixtures of drug substances and excipient(s) (3, 4) resulted in minimal formation of *N*-methyl and *N*-formyl degradation products (Figure 6). On the contrary, separated configuration yielded substantial amounts of impurities, while the drug substance decrease remained low. PEG alone in a separate configuration (5), produced some amine-functionality-related degradation, but this was not the case for all drug substances (Figure 6a). PEG combined with Fe_2_O_3_ induced degradation of all seven compounds. Compared to other compatibility setups, PEG combined with Fe_2_O_3_ in a separate setup (6) produces the highest sum of *N*-methyl and *N*-formyl degradation across all drug substances (Figure 6b).

**Figure 7:**
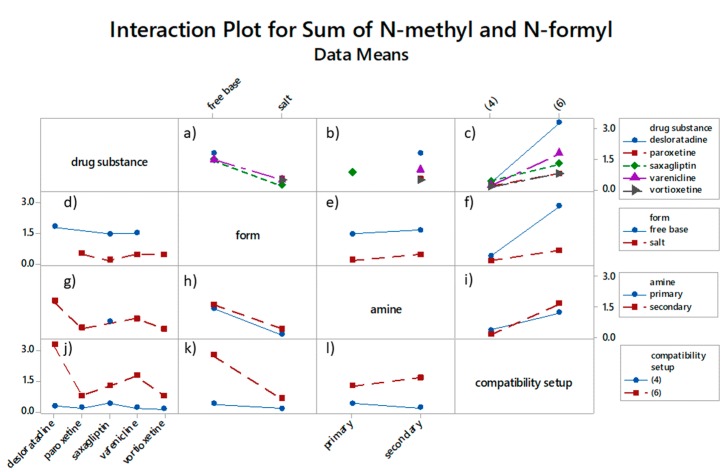
Interaction plot for sum of *N*-methyl and *N*-formyl impurities for multiple factors (grouped), based on data means. Data grouped by ‘drug substance’: ‘substance form’ on x axis (**a**), ‘type of amine’ on x axis (**b**), ‘compatibility setup’ on x axis (**c**); data grouped by ‘substance form’: drug substance on x axis (**d**), ‘type of amine’ on x axis (**e**), ‘compatibility setup’ on x axis (**f**); data grouped by ‘type of amine’: ‘drug substance’ on x axis (**g**), ‘substance form’ on x axis (**h**), ‘compatibility setup’ on x axis (**i**); data grouped by ‘compatibility setup’: drug substance on x axis (**j**), ‘substance form’ on x axis (**k**), ‘type of amine’ on x axis (**l**). Only tertiary design data from Table 6 is used for analysis.

Based on the data from tertiary designs, compatibility setup (4) and (6), the reactivity of amine-functionality-containing drugs with PEG degradation products is strongly affected by whether the substance is in free base or salt form (Figure 7a,d). The former is true for primary and secondary amines (Figure 7e,h). In separated design, free base forms are on average more than three times more reactive than salts (Figure 7k,f). On the other hand, secondary amine compounds are on average slightly more reactive than primary amine compounds, in free base as in salt form (Figure 7e,h). This is true only for separate design, however, there the degradation is much more pronounced, therefore making this setup more relevant (Figure 7i,l). Higher observed reactivity of secondary amines is ascribed to the greater nucleophilic potential of these compounds [25].

The low formation of *N*-methyl varenicline in samples where varenicline is in free base form is ascribed to a high formic acid consumption for the *N*-formylation reaction. The following is reflected in a very high *N*-formyl to *N*-methyl impurity ratio (Table 7). The *N*-formylation reaction has been shown to have high activation energy (activation energy of 151.6 kJ per mole was determined for saxagliptin *N*-formylation in our previous study [15]). Free base forms of secondary amines, as the most reactive of tested compounds, are capable of overcoming this barrier, and readily react with formic acid (ratio *N*-FOR vs. *N*-MET above 1). On the other hand, free base forms of primary amines are less reactive (ratio *N*-FOR vs. *N*-MET below 1). The high energy barrier of *N*-formylation reaction steers the amine groups to react with formaldehyde in the *N*-methylation reaction. In agreement with this is the case of primary amine saxagliptin in salt form, which was found to be the least reactive with PEG degradation products within the tested set of molecules. For saxagliptin in salt form, the formation of *N*-formyl impurities was close to zero. Potential parallel and consecutive degradation pathways that could influence *N*-formyl and *N*-methyl levels were not considered for any of the compounds. An extended pool of amine-functionality-containing drugs would be needed to generalize our conclusions further, also in view of the effects that different salts have on the amine functionality reactivity.

## 4. Conclusions

This paper elaborated a new stressing procedure that is capable of identifying *N*-formylation and *N*-methylation susceptible amine-functionality-containing drugs using excipients in actual ratios from the solid dosage form. The proposed approach is more realistic, selective, and robust compared to an approach where spiking with reactive impurities is used. Through a kinetic study of polyethylene glycol degradation, we established that the presence of iron oxides in film-coated tablet formulation is responsible for elevated levels of formic acid by increasing rate constants for PEG to formaldehyde and formaldehyde to formic acid degradation. The *N*-formyl to *N*-methyl impurity ratio was associated with the overall amine functionality reactivity and concentration of formed formaldehyde and formic acid in the samples. The most reactive drug substance tested, secondary amines in free base form, were more likely to form *N*-formyl impurities, whereas the least reactive substance, primary amines in salt form, were more likely to form *N*-methyl impurities.

## Figures and Tables

**Table 1:** Compounds’ *m/z* used for selected ion monitoring (SIM) acquisitions using electrospray ionization in positive mode ((+)ESI-MS).

*m/z*	Paroxetine	Desloratadine	Vortioxetine	Varenicline	Saxagliptin
Drug substance	330	311	299	212	316
*N*-Methyl impurity	344	325	313	226	330
*N*-Formyl impurity	358	339	327	240	344

**Table 2:** (+)ESI-MS conditions optimized for individual drug substances.

MS Tune Conditions	Units	Paroxetine	Desloratadine	Vortioxetine	Varenicline	Saxagliptin
Heater temperature	°C	50	50	50	50	50
Sheath gas flow rate	a.u.	10	30	30	22	28
Auxiliary gas flow rate	a.u.	10	10	10	2	5
Sweep gas flow rate	a.u.	0	0	0	0	0
Spray voltage	kV	5	5	5	5	5
Capillary temperature	°C	300	300	300	250	250
Capillary voltage	V	34.41	20.41	29.41	32	49
Tube lens	V	97	102	83	90	110

Abbreviations: °C: Degrees Celsius; a.u.: Arbitrary units; V: Volts.

**Table 3:** Origin of reactive impurities in the tablet formulation. Paroxetine hydrochloride (200 mg) alone or mixed with components from formulation was weighed into glass vials in ratios defined in the table (first value indicates drug substance, second component 1 and third component 2) and sealed. Mixtures of excipient components are in direct contact or separate from the drug substance (compatibility setup). The *N*-methyl paroxetine (*N*-MET) percentage (%) presents the area % of the determined impurity after seven days at 60 °C.

Experiment	Component 1	Component 2	Ratio	Compatibility Setup	*N*-MET [%]
1	-	-	1	-	0.05
	PVP K30	-	1:1	direct	0.05
	PEG 6000	-	1:0.1	direct	0.05
	TEC	-	1:0.1	direct	0.05
2	PEG 6000	Fe_2_O_3_ yellow	1:0.1:0.01	direct	0.05
	PEG 6000	-	1:0.1	separate	0.04
	PEG 6000	Fe_2_O_3_ yellow	1:0.1:0.01	separate	0.63
3	TEC	Fe_2_O_3_ yellow	1:0.1:0.01	separate	0.05
	PEG 6000	Quinoline yellow lake	1:0.1:0.01	separate	0.05

Abbreviations: Povidone (PVP); polyethylene glycol (PEG); triethyl citrate (TEC); and iron oxide (Fe_2_O_3_).

**Table 4:** *N*-methyl impurity formation with a direct reactive impurity spike. Paroxetine hydrochloride (50 mg) was weighed into glass vials to which 10 µL of stressing liquid was added. The exact molar ratios are 1:0.985 (formaldehyde) and 1:1.94 (formic acid). The *N*-methyl paroxetine (*N*-MET) and drug substance [%] presents area % of the concerning peak after seven days at 60 °C in closed vials.

Reactive Impurity	Compatibility Setup	Drug Substance [%]	*N*-MET [%]
Formaldehyde	direct	89.3	1.02
Formaldehyde	separate	71.7	0.98
Formic acid	direct	93.0	0.03
Formic acid	separate	94.9	0.09

**Table 5:** Evaluation of different factors on the formation of % *N*-methyl paroxetine. Paroxetine hydrochloride alone or mixed with components from the formulation was weighed into glass vials in the ratios defined in the table (the first value indicates the drug substance, the second indicates PEG 6000, the third indicates Fe_2_O_3_, and the fourth indicates the additive). Mixtures of excipient components were separate from the drug substance. Additives were added to the excipient mixture. 10 µL additions of water, 0.1 M hydrochloride acid, and 0.1 M sodium hydroxide were evaluated. The *N*-methyl paroxetine (*N*-MET) percentage (%) presents an area % of the determined impurity after seven days at 60 °C in closed vials, except control experiment which was done in open-dish conditions.

Atmosphere/Additive	Ratio	*N*-MET [%]
-	1:0.1:0.01	0.63
-	1:0.2:0.02	0.79
-	1:0.1:0.05	0.77
-	1:0.1:0.005	0.13
HPMC	1:0.1:0.01:1	0.04
H_2_O	1:0.1:0.01	0.04
H^+^	1:0.1:0.01	0.04
OH^-^	1:0.1:0.01	0.04
N_2_	1:0.1:0.01	0.04
Open-dish	1:0.1:0.01	0.04

Abbreviations: Polyethylene glycol (PEG); iron oxide (Fe_2_O_3_); hydroxypropyl methyl cellulose (HPMC).

**Table 6:** Seven model compounds with amine functionality tested by LC-MS for polyethylene glycol (PEG) incompatibility. The drug substance alone or with added formaldehyde/excipients was subjected to stress testing at 60 °C for seven days in closed glass vials. A weight ratio of 1:0.1:0.01 for paroxetine·HCl, PEG, and Fe_2_O_3_ was transcribed into molar concentrations, and the same molar ratios were used for other drug substances. Formaldehyde was added as formalin (10 µL), presenting a molar ratio of 1:0.985. Mixtures of excipient components were in direct contact or separate from the drug substance (compatibility setup). The *N*-methyl paroxetine (*N*-MET) and *N*-formyl (*N*-FOR) percentages (%) present moles of impurity against the introduced moles of the drug substance in the vial after seven days at 60 °C.

Drug Substance	#	Additive	Compatibility Setup	*N*-MET [%]	*N*-FOR [%]
Paroxetine·HCl	(1)	-	-	n.d.	n.d.
	(2)	F	separate	3.19	0.47
	(3)	PEG	direct	<loq	<loq
	(4)	PEG + Fe_2_O_3_	direct	<loq	n.d.
	(5)	PEG	separate	<loq	n.d.
	(6)	PEG + Fe_2_O_3_	separate	**0.67**	**<loq**
Vortioxetine·HBr	(1)	-	-	n.d.	n.d.
	(2)	F	separate	1.82	0.13
	(3)	PEG	direct	n.d.	n.d.
	(4)	PEG + Fe_2_O_3_	direct	n.d.	n.d.
	(5)	PEG	separate	0.37	n.d.
	(6)	PEG + Fe_2_O_3_	separate	**0.67**	**<loq**
Varenicline tartrate	(1)	-	-	n.d.	n.d.
	(2)	F	separate	7.78	1.09
	(3)	PEG	direct	n.d.	n.d.
	(4)	PEG + Fe_2_O_3_	direct	n.d.	0.07
	(5)	PEG	separate	0.19	n.d.
	(6)	PEG + Fe_2_O_3_	separate	**0.62**	**<loq**
Varenicline base	(1)	-	-	n.d.	n.d.
	(2)	F	separate	19.8	10.8
	(3)	PEG	direct	n.d.	<loq
	(4)	PEG + Fe_2_O_3_	direct	n.d.	<loq
	(5)	PEG	separate	<loq	2.21
	(6)	PEG + Fe_2_O_3_	separate	**0.07**	**2.71**
Saxagliptin·HCl	(1)	-	-	n.d.	n.d.
	(2)	F	separate	36.0	0.27
	(3)	PEG	direct	n.d.	n.d.
	(4)	PEG + Fe_2_O_3_	direct	n.d.	n.d.
	(5)	PEG	separate	0.08	n.d.
	(6)	PEG + Fe_2_O_3_	separate	**0.15**	**n.d.**
Saxagliptin base	(1)	-	-	n.d.	n.d.
	(2)	F	separate	4.32	0.41
	(3)	PEG	direct	n.d.	0.20
	(4)	PEG + Fe_2_O_3_	direct	n.d.	0.59
	(5)	PEG	separate	1.03	0.63
	(6)	PEG + Fe_2_O_3_	separate	**1.32**	**0.95**
Desloratadine base	(1)	-	-	0.15	n.d.
	(2)	F	separate	3.89	0.05
	(3)	PEG	direct	0.20	n.d.
	(4)	PEG + Fe_2_O_3_	direct	0.20	n.d.
	(5)	PEG	separate	0.16	n.d.
	(6)	PEG + Fe_2_O_3_	separate	**0.95**	**2.38**

Abbreviations: Not detected (n.d.); below limit of quantification (<loq); hydrochloride (HCl); hydrobromide (HBr); polyethylene glycol (PEG); and iron oxide (Fe_2_O_3_).

**Table 7:** Seven model compounds with amine functionality tested for polyethylene glycol (PEG) incompatibility. Two presented parameters were calculated based upon the results of samples of drug substances in combination with PEG and iron oxide (Fe_2_O_3_) in separate vials after seven days at 60 °C in closed vials (data from Table 6 used).

Drug Substance	Sum of *N*-MET and *N*-FOR (%)	Ratio of *N*-FOR vs. *N*-MET
Paroxetine·HCl	0.74	0.09
Vortioxetine·HBr	0.73	0.09
Varenicline tartrate	0.65	0.05
Varenicline base	2.77	41.1
Saxagliptin·HCl	0.15	0.00
Saxagliptin base	2.27	0.72
Desloratadine base	3.33	2.50

Abbreviations: *N*-methyl impurity (*N*-MET) and *N*-formyl impurity (*N*-FOR); hydrochloride (HCl); hydrobromide (HBr).

## Supplementary Materials

The following are available online at https://www.mdpi.com/1999-4923/12/1/37/s1, Figure S1: IR spectrum of paroxetine *N*-formyl impurity, Figure S2: IR spectrum of vortioxetine *N*-methyl impurity, Figure S3: IR spectrum of saxagliptin *N*-methyl impurity, Figure S4: MS spectra of paroxetine and its *N*-methyl- and *N*-formyl- impurities (bottom left), paroxetine samples 2 and 6 full scan (top left) and SIM chromatograms of standards and some samples (right), Figure S5: MS spectra of desloratadine and its *N*-methyl- and *N*-formyl- impurities (bottom left), desloratadine sample 6 full scan (top left) and SIM chromatograms of standards and some samples (right), Figure S6: MS spectra of vortioxetine and its *N*-methyl- and *N*-formyl- impurities (bottom left), vortioxetine sample 6 full scan (top left) and SIM chromatograms of standards and some samples (right), Figure S7: MS spectra of varenicline and its *N*-methyl- and *N*-formyl- impurities (bottom left), varenicline base and tartrate samples 6 full scan (top left) and SIM chromatograms of standards and some samples (right), Figure S8: MS spectra of saxagliptin and its *N*-methyl- and *N*-formyl- impurities (bottom left), saxagliptin base and HCl samples 6 full scan (top left) and SIM chromatograms of standards and some samples (right), Table S1: Summarized method performance parameters.
