# Supplementary material for: A Novel Testing Approach for Oxidative Degradation Dependent Incompatibility of Amine Moiety Containing Drugs with PEGs in Solid-State
Source: Pharmaceutics. 2020 Jan 2;12(1):37. doi: 10.3390/pharmaceutics12010037 (PMC7022946; doi:10.3390/pharmaceutics12010037)
Supplement: Supplementary file 1 [file pharmaceutics-12-00037-s001.pdf]

pharmaceutics

Supplementary Materials: A Novel Testing Approach for Oxidative Degradation Dependent Incompatibility of Amine Moiety Containing Drugs with PEGs in Solid-State

Blaž Robnik, Katerina Naumoska and Zdenko Časar

1. Analytical Methods

1.1. HPLC-MS Molecular Weight Confirmation of Synthesized Standards

Synthesized standards were analyzed using Agilent 1200 series HPLC system (Santa Clara, CA, US), on a reverse phase column, Luna C18(2), (100 × 4.6 mm; 3 µm, Phenomenex Inc., Torrance, CA, US). Gradient elution was used to achieve chromatographic separation with mobile phases A (0.2% trifluroacetic acid in water), B (1% acetonitrile in water) and C (acetonitrile). The % of mobile phase A was kept constant (5%) during the gradient elution program, while other parameters were as follows: 5%–85% C (0–20 min), 85% C (20–25 min), 5% C (25.1–35.1 min). The mobile phase flow rate was 1.1 mL/min. Autosampler and column temperatures were set at 5 °C and 40 °C, respectively. The injection volume was 5 µL. Mass spectrometer Bruker HCT (Billerica, MA, US) was used for detection in positive electrospray ionization mode. Scanning range was set to 50–1000 *m/z*. Other MS parameters were as follows: nebulizing gas 35 psi, drying gas 8 L/min and drying temperature 300 °C.

1.2. UHPLC-MS Determination of Drug Substance, N-Methyl and N-Formyl Degradation Products: Method Performance Parameters/Method Verification

Limit of quantification (LOQ), limit of detection (LOD), % relative standard deviation (RSD) calculated for areas (n = 6) at 25 µg/mL for drug substances and at 187.5 ng/mL for *N*-methyl and *N*-formyl impurities, R^2, range, accuracy and precision were calculated and are summarized in Table S1. Accuracy was determined as % of the recovered amount calculated based on the calibration curve versus added amount (n = 6) at 25 µg/mL for drug substances and at 187.5 ng/mL for *N*-methyl and *N*-formyl impurities. Precision was calculated as % RSD (areas) measured using the samples which correspond to compatibility setups (6) prepared in six replicates (to calculate precision for saxagliptin, varenicline and their corresponding impurities, saxagliptin HCl and varenicline base samples were prepared, respectively). Results reported in Table 7 include also values between LOD and LOQ, to enable for calculation of presented ratios.

Table S1. Summarized method performance parameters.

	LOQ [ng/mL]	LOD [ng/mL]	RSD [%]	R^2	Range	Accuracy (%), ($RSD_{ACCURACY}$ [%])	Precision (RSD [%])
Paroxetine	-	-	1.80	0.9968	5–35 µg/mL	95 (2.8)	1.0
N-Methyl paroxetine	23.4	5.8	2.37	0.9998	23.4–3000 ng/mL	104 (2.5)	2.8
N-Formyl paroxetine	23.4	5.8	3.22	0.9999	23.4–3000 ng/mL	95 (3.1)	4.0
Desloratadine	-	-	2.01	0.9987	5–35 µg/mL	105 (3.3)	2.3
N-Methyl desloratadine	23.4	11.7	1.85	1	23.4–3000 ng/mL	101 (1.7)	1.5

N-Formyl desloratadine	11.7	5.8	1.95	0.9996	11.7–3000 ng/mL	104 (4.1)	2.5
Vortioxetine	-	-	2.11	0.996	5–35 µg/mL	101 (3.3)	3.1
N-Methyl vortioxetine	23.4	11.7	4.17	0.9999	23.4–3000 ng/mL	96 (3.8)	4.3
N-Formyl vortioxetine	23.4	11.7	2.27	0.9999	23.4–3000 ng/mL	101 (2.2)	4.2
Varenicline	-	-	1.6	0.9939	5–35 µg/mL	97 (2.1)	1.9
N-Methyl varenicline	23.4	5.8	2.4	0.9998	23.4–3000 ng/mL	97 (2.4)	3.8
N-Formyl varenicline	11.7	5.8	4.2	0.9999	11.7–3000 ng/mL	93 (4.3)	1.2
Saxagliptin	-	-	3.6	0.9984	5–35 µg/mL	103 (3.7)	2.7
N-Methyl saxagliptin	11.7	5.8	2	0.9996	11.7–3000 ng/mL	104 (2.1)	3.7
N-Formyl saxagliptin	46.9	23.4	2.8	0.9992	46.9–3000 ng/mL	96 (3.6)	/ (n.d.)

1.3. UHPLC-UV Determination of Paroxetine Degradation Products

For determination of paroxetine drug substance and *N*-methyl paroxetine impurity, supplementary analytical method, running on Acquity UPLC system (Waters Corp., Milford, MA, US) was used (results in 3.1. PEG incompatibility stress test: paroxetin·HCl). A reverse phase column Acquity BEH C18, (100 × 2.1 mm; 1.7 µm, Waters Corp., Milford, MA, USA) and a gradient elution were employed to achieve chromatographic separation. Mobile phase A consisted of ammonium buffer with pH 10.25 and mobile phase B contained water:acetonitrile, 10:90 (*v*/*v*). Gradient program was as follows: 35%B (0–0.5 min), 35%–45%B (0.5–7min), 45%–75%B (7–9min), 75%B (9–10.5 min), 75%–35% (10.5–11min). After each run, re-equilibration to initial conditions was performed. The mobile phase flow rate was 0.55 mL/min. Autosampler and column temperatures were set at 5 °C and 40 °C, respectively. The injection volume was 7 µL. Data was recorded by photo diode array (PDA) detector at a wavelength of 295 nm. Aged samples of paroxetine·HCl alone or as mixtures with excipients in glass vials were dissolved in 50 mL of solvent (mixture of acetonitrile, methanol and acetic buffer, pH 4.5). Solutions were sonicated and filtered prior to injection. Results were calculated as area % of *N*-methyl paroxetine against paroxetine.

2. Characterization of standards

2.1. NMR Measurement Data (Chemical Shifts and Coupling Constants) for Synthesized Standards

2.1.1 *N*-Formyl Paroxetine

^{1}H NMR (600 MHz, Chloroform-*d*) δ 8.14 (s, 1H), 8.12 (s, 0H), 7.13 (ddt, *J* = 6.8, 5.3, 1.6 Hz, 2H), 7.05 – 6.93 (m, 2H), 6.63 (dd, *J* = 8.5, 5.3 Hz, 1H), 6.36 (dd, *J* = 3.9, 2.5 Hz, 1H), 6.14 (dt, *J* = 8.5, 2.6 Hz, 1H), 5.94 – 5.84 (m, 2H), 4.73 (ddd, *J* = 13.5, 4.4, 1.7 Hz, 0H), 4.58 (ddt, *J* = 13.3, 4.3, 1.9 Hz, 1H), 4.12 (q, *J* = 7.1 Hz, 1H), 3.97 (ddd, *J* = 13.5, 4.3, 1.8 Hz, 1H), 3.74 (dtt, *J* = 11.1, 4.8, 2.4 Hz, 1H), 3.62 (ddd, *J* = 9.4, 8.3, 2.9 Hz, 1H), 3.53 – 3.41 (m, 1H), 3.22 (td, *J* = 13.0, 3.0 Hz, 0H), 3.15 (dd, *J* = 13.3, 11.3 Hz, 1H), 2.87 (td, *J* = 11.9, 3.8 Hz, 1H), 2.81 – 2.68 (m, 2H), 2.02 – 2.10 (m, 2H), 2.01 – 1.82 (m, 2H), 1.76 – 1.64 (m, 1H), 1.26 (t, *J* = 7.1 Hz, 2H).

^{13}C NMR (151 MHz, Chloroform-*d*) δ 171.15, 161.69 (d, *J* = 245.4 Hz), 160.93, 160.85, 154.13, 153.90, 148.26, 148.17, 141.90, 141.78, 138.31 (d, *J* = 3.3 Hz), 138.19 (d, *J* = 3.3 Hz), 128.71 (d, *J* = 7.9 Hz), 128.62 (d, *J* = 7.7 Hz), 115.86 – 115.63 (m), 115.58, 107.89, 107.84, 105.56, 105.46, 101.19, 101.12, 97.99, 97.87, 68.59, 68.39, 60.38, 49.21, 46.22, 44.50, 44.06, 42.89, 42.86, 41.52, 40.12, 34.54, 33.16, 21.04, 14.18.

2.1.2. *N*-Methyl Vortioxetine

^{1}H NMR (399 MHz, Chloroform-*d*) δ 7.36 (d, *J* = 7.8 Hz, 1H), 7.16 (d, *J* = 1.8 Hz, 1H), 7.09 (dd, *J* = 5.0, 1.2 Hz, 2H), 7.04 (dd, *J* = 7.6, 2.0 Hz, 1H), 6.95 – 6.84 (m, 1H), 6.54 – 6.47 (m, 1H), 3.34 – 3.19 (m, 4H), 3.01 (s, 4H), 2.68 (s, 3H), 2.36 (s, 3H), 2.31 (s, 3H).

^{13}C NMR (100 MHz, Chloroform-*d*) δ 147.77, 142.39, 139.47, 136.18, 134.49, 131.75, 127.89, 127.34, 126.14, 125.66, 125.09, 120.14, 55.42, 50.13, 45.36, 21.19, 20.58.

2.1.3. *N*-Methyl saxagliptin

^{1}H NMR (399 MHz, Methanol-d_4) δ 5.48 (s, 0H), 5.18 – 4.98 (m, 1H), 3.96 – 3.75 (m, 1H), 3.58 (s, 0H), 3.37 (s, 1H), 3.23 (d, J = 3.2 Hz, 0H), 2.70 – 2.54 (m, 1H), 2.37 – 2.27 (m, 1H), 2.26 (s, 2H), 2.24 – 2.12 (m, 2H), 2.03 – 1.74 (m, 4H), 1.72 – 1.63 (m, 4H), 1.63 – 1.53 (m, 3H), 1.52 – 1.41 (m, 3H), 1.18 – 1.10 (m, 2H), 1.10 – 1.01 (m, 1H), 0.98 – 0.89 (m, 1H).

^{13}C NMR (100 MHz, cd_3od) δ 174.55, 174.36, 120.88, 120.85, 70.53, 69.23, 69.16, 61.23, 47.30, 46.74, 46.53, 46.39, 45.40, 45.37, 45.30, 45.26, 42.20, 41.92, 39.19, 38.98, 38.78, 38.72, 38.61, 38.25, 36.65, 36.60, 35.21, 31.90, 31.85, 31.79, 31.77, 31.48, 31.46, 18.85, 18.76, 14.11, 14.00.

2.2. IR Spectra for Synthesized Standards

2.2.1 N-Formyl Paroxetine

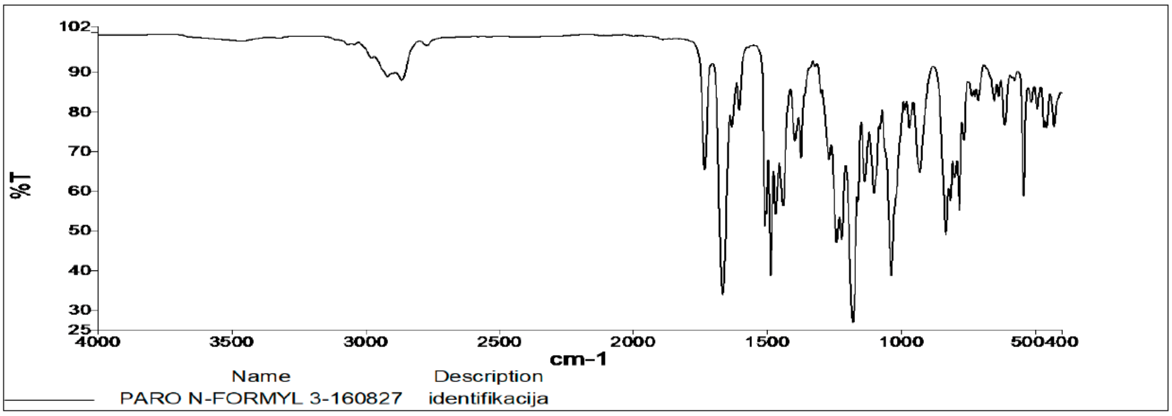

Figure S1. IR spectrum of paroxetine *N*-formyl impurity.

2.1.2. N-Methyl vortioxetine

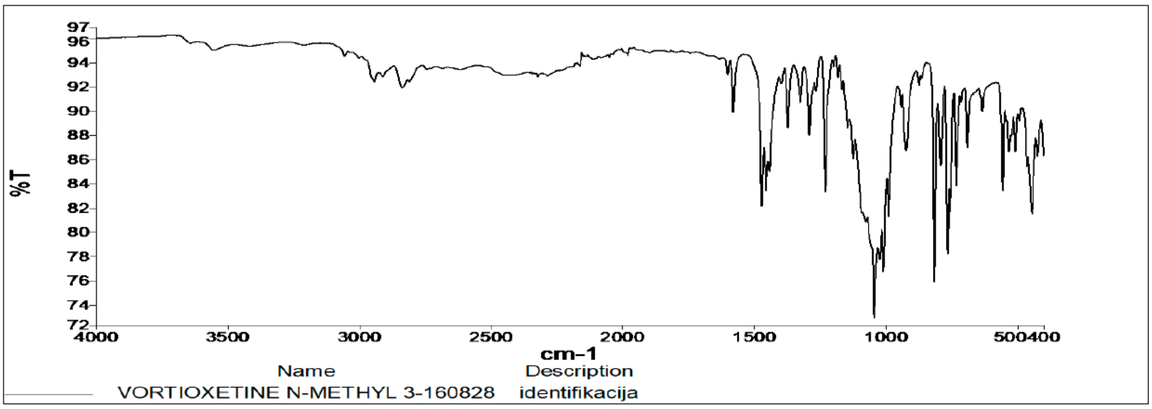

Figure S2. IR spectrum of vortioxetine *N*-methyl impurity.

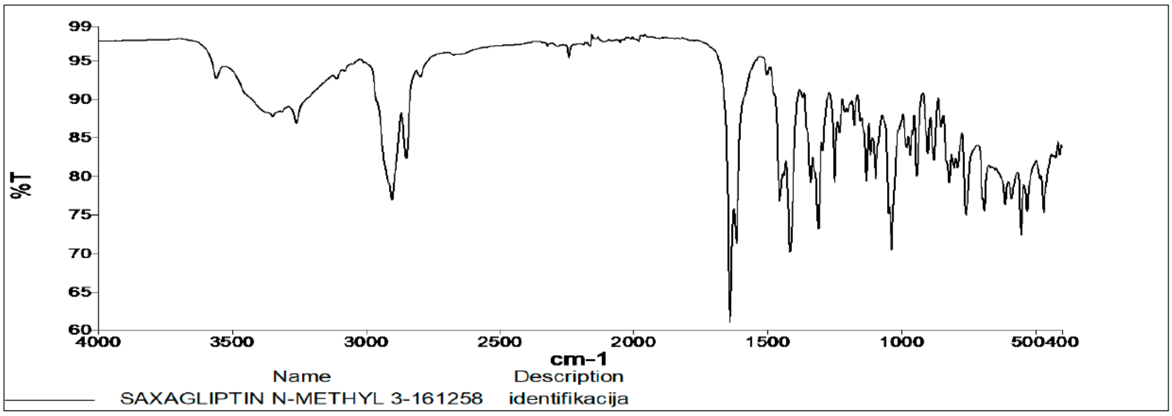

Figure S3. IR spectrum of saxagliptin *N*-methyl impurity.

2.3. LC-MS Chromatograms and MS Spectra

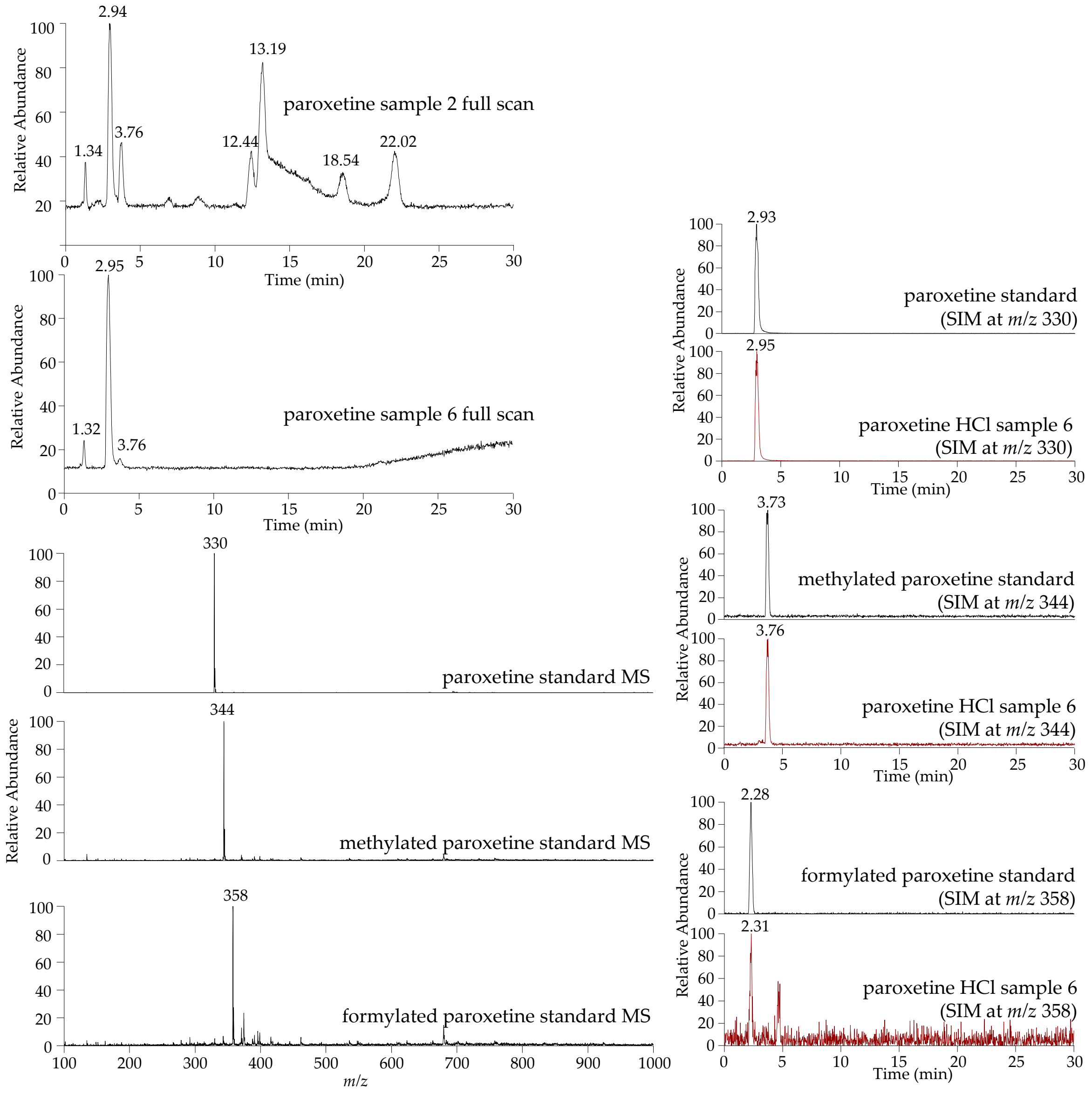

Figure S4. MS spectra of paroxetine and its *N*-methyl- and *N*-formyl- impurities (bottom left), paroxetine samples 2 and 6 full scan (top left) and SIM chromatograms of standards and some samples (right).

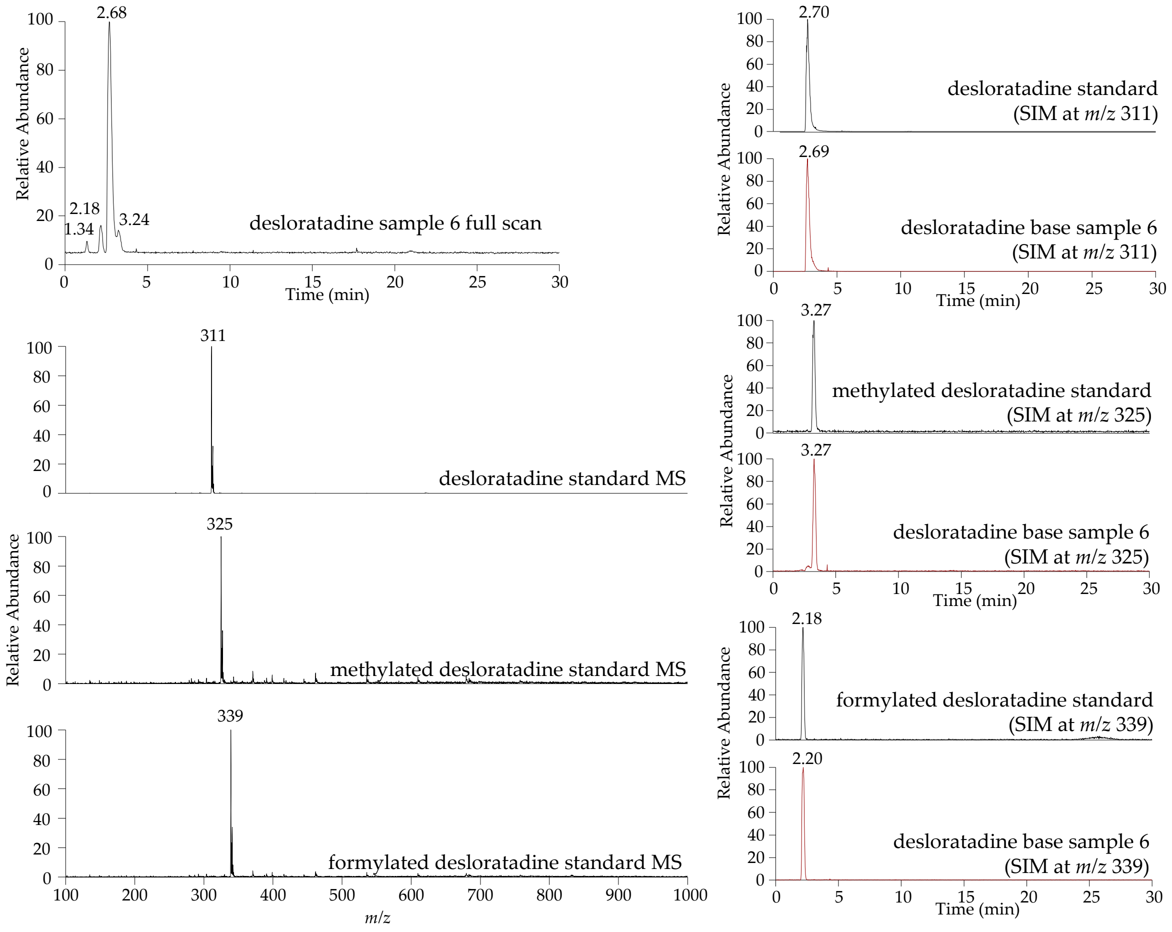

Figure S5. MS spectra of desloratadine and its *N*-methyl- and *N*-formyl- impurities (bottom left), desloratadine sample 6 full scan (top left) and SIM chromatograms of standards and some samples (right).

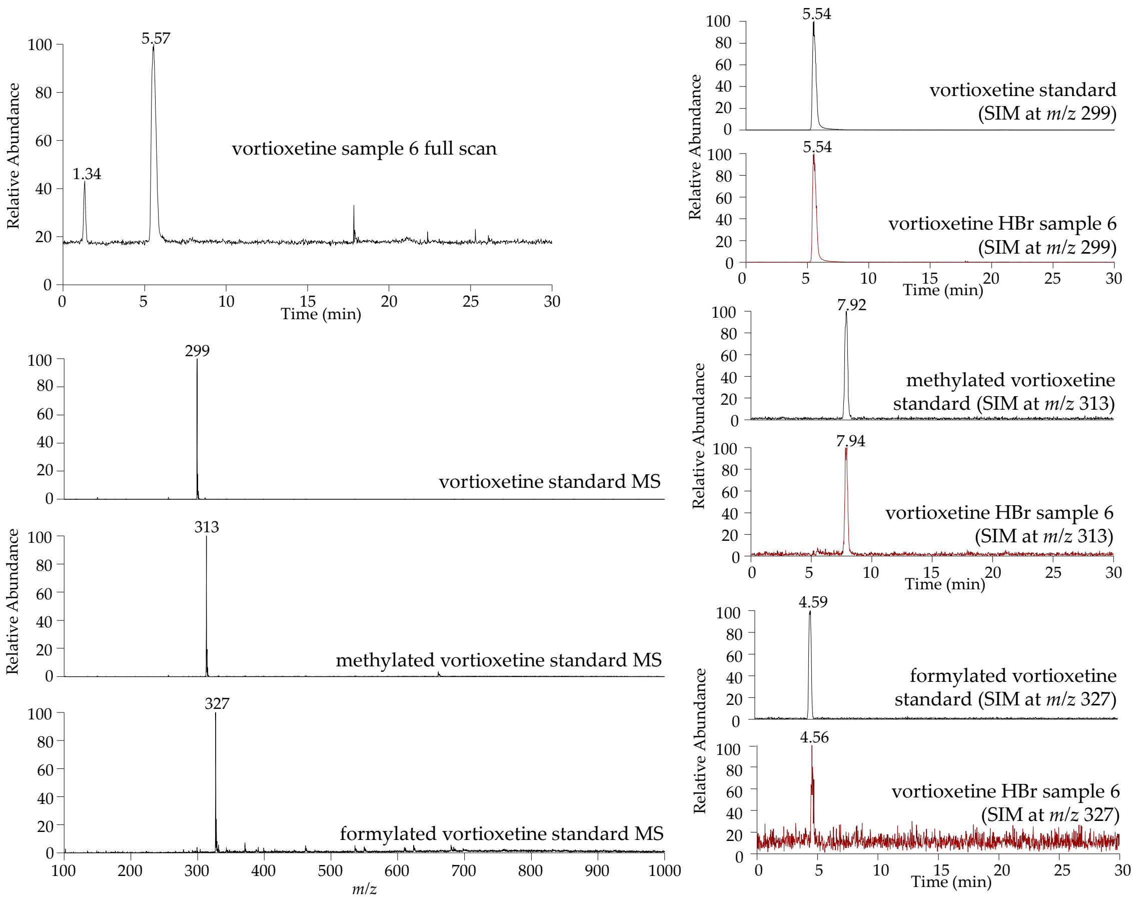

Figure S6. MS spectra of vortioxetine and its *N*-methyl- and *N*-formyl- impurities (bottom left), vortioxetine sample 6 full scan (top left) and SIM chromatograms of standards and some samples (right).

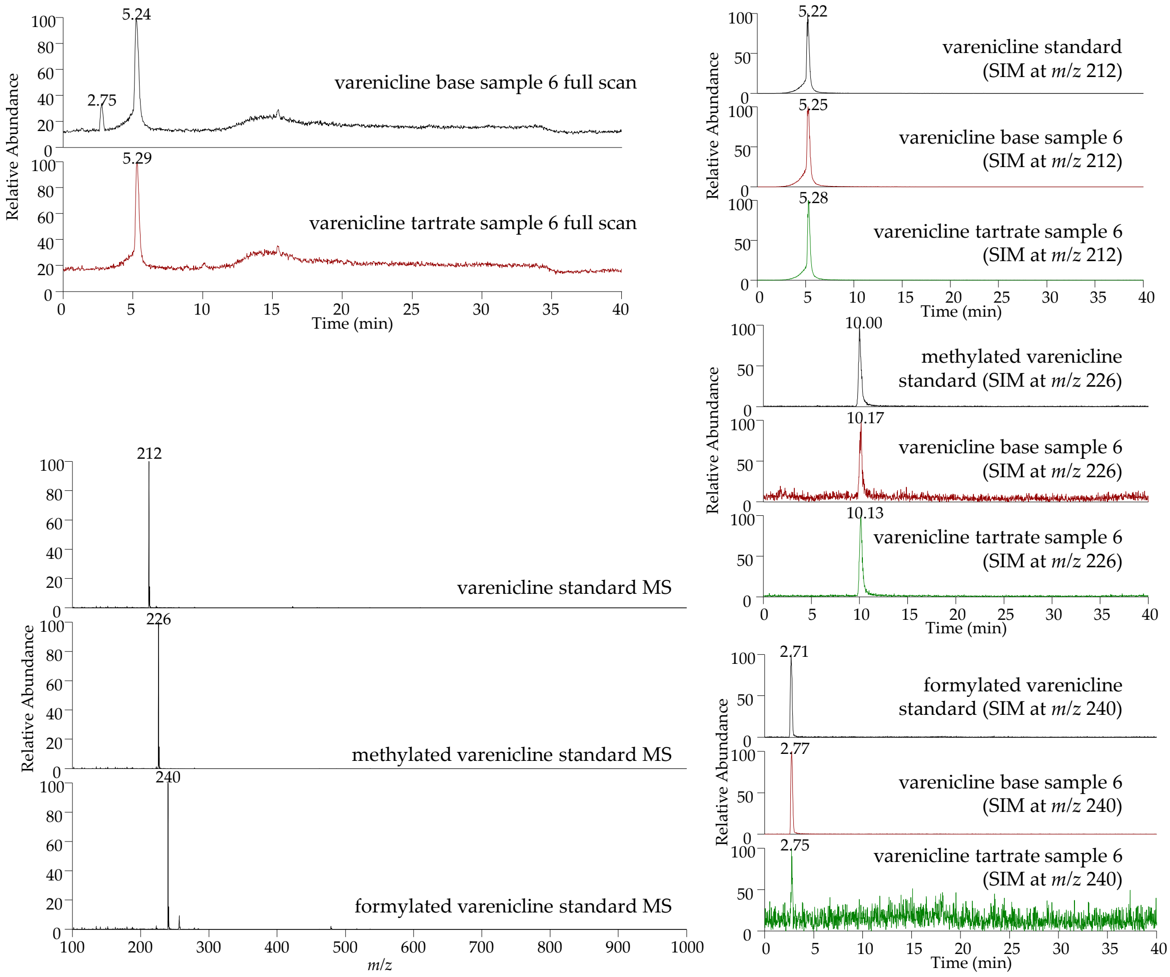

Figure S7. MS spectra of varenicline and its *N*-methyl- and *N*-formyl- impurities (bottom left), varenicline base and tartrate samples 6 full scan (top left) and SIM chromatograms of standards and some samples (right).

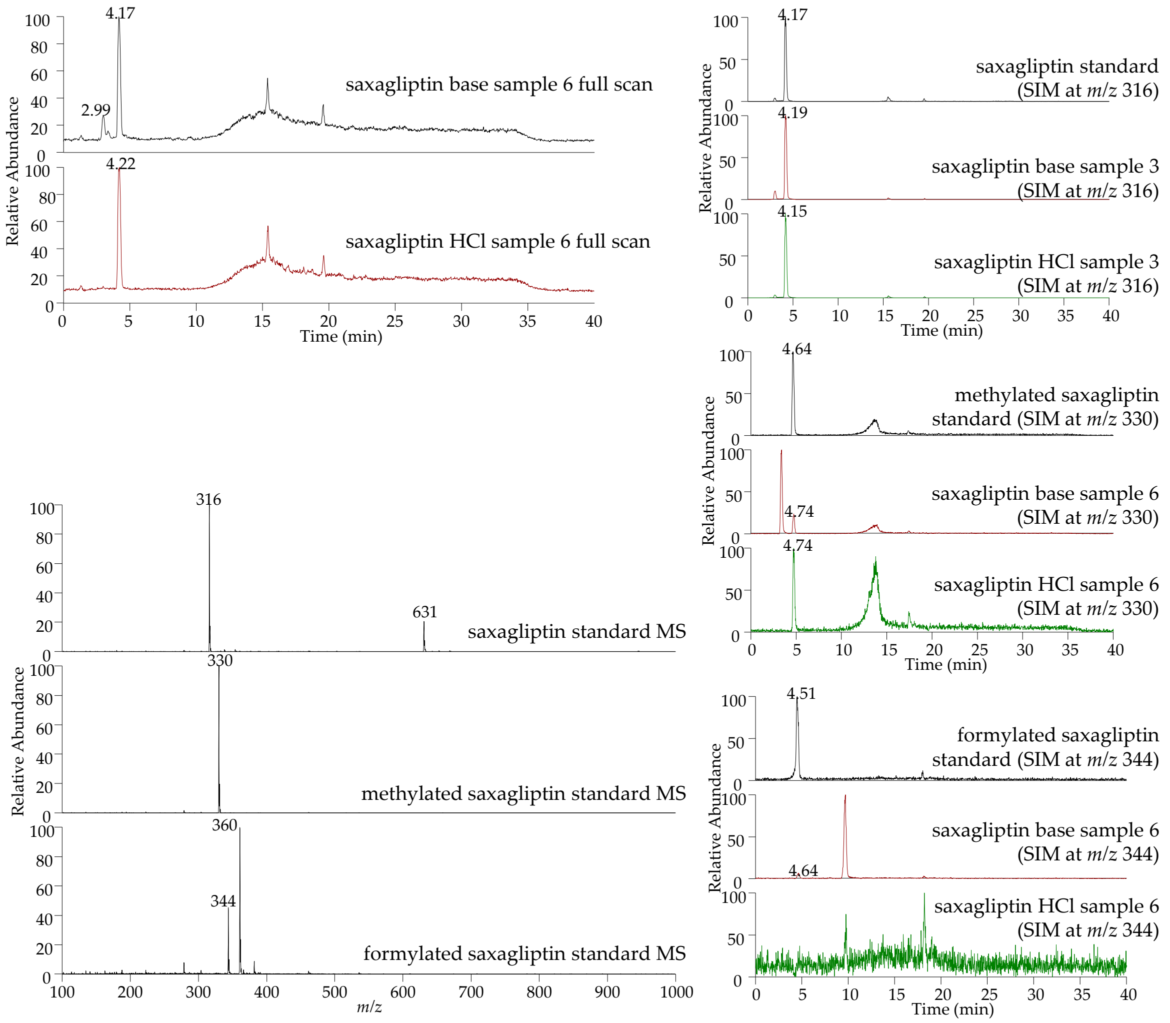

Figure S8. MS spectra of saxagliptin and its *N*-methyl- and *N*-formyl- impurities (bottom left), saxagliptin base and HCl samples 6 full scan (top left) and SIM chromatograms of standards and some samples (right).

© 2020 by the author. Licensee MDPI, Basel, Switzerland. This article is an open access article distributed under the terms and conditions of the Creative Commons Attribution (CC BY) license (http://creativecommons.org/licenses/by/4.0/).
